# Association between religiosity or spirituality and internet addiction: A systematic review

**DOI:** 10.3389/fpubh.2022.980334

**Published:** 2022-12-01

**Authors:** Francesca Dossi, Alessandra Buja, Laura Montecchio

**Affiliations:** Department of Cardiologic, Vascular and Thoracic Sciences, and Public Health, University of Padua, Padua, Italy

**Keywords:** religiosity, spirituality, internet addiction, internet gaming addiction, internet gambling addiction

## Abstract

**Introduction:**

The literature provides evidence of religiosity being associated with physical and mental health, and also with behavioral addictions. This systematic review examines the data on the link between religiosity or spirituality and the emerging internet addictions.

**Methods:**

A systematic literature review was conducted in the PubMed and Scopus databases to identify observational (cross-sectional, cohort, and case-control) studies conducted on adolescents and young adults to investigate the association between religiosity or spirituality and internet addiction. Of the 854 articles identified in the databases, 13 met our inclusion criteria and were included in our systematic review.

**Results:**

Eleven of the 13 studies reviewed specifically investigated religiosity and internet addiction: six found an inverse association between religiosity and internet addiction; three found no evidence of any association; and one found a direct association. One study examining both religiosity and spirituality generated mixed results. Only one study investigated spirituality unrelated to religion, and found a direct association with internet addiction. Two of three studies specifically considering internet gaming addiction found it inversely associated with high levels of religiosity, while the third found no association.

**Conclusion:**

This review supports a possible role for religiosity as a protective factor, as emerged from the majority of the studies examined. Religiosity also seemed to be associated with lower internet gaming rates among adolescents.

## Introduction

There is evidence in the literature of both religiosity and spirituality being associated with health status (1), and empirical data indicate that specific aspects of religiosity relate to mental health (2).

Religiosity can be defined as “the self-perceived importance of religion and the degree to which religious beliefs and identities translate into secular attitudes” (3), and includes aspects related to being part of a community who share the same values and interests; spirituality may be seen as an aspect that can be experienced both outside and inside a religious context and characterized by a desire for transcendence, a sense of interconnection and a meaningful sense of life (4). Both religiosity and spirituality could lend a deep sense of purpose in one's own existence, reduce stress, and suggest coping mechanisms (5, 6). Much research in recent years has focused on the role that religiosity and spirituality might have in people's vulnerability to addiction, including behavioral addictions (7, 8). Among the latter, those relating to internet use represent a growing problem (9, 10), especially in adolescents but also in young adults (11), with students accounting for the majority of the latter (12). Various studies have identified internet addiction (IA) as an international phenomenon with an overall prevalence estimated to be around 4.6–4.7% for adolescents, and in the range of 13–18.4% for college students, as compared with 6–15% for the general population (13). Over the years, researchers have described this addiction as a syndrome of intense preoccupation with using the internet (14), involving excessive amounts of time spent online, a compulsive use of the internet, difficulty with managing the time spent on the internet, feeling that the world outside the internet is boring, becoming irritated if disturbed while online, reducing social interaction with “real” people (15), and tried by an experience of loneliness and depression (16). Despite the growing body of research into IA over the last decade, there is still no widely-accepted definition of this condition. People can engage in various activities on the web, some of which are potentially addictive. Young (17) argues that IA could take different forms, such as: information overload (web surfing addiction); computer addiction (to computer games); net compulsions (addiction to online gambling, or online shopping); cyber-sexual addiction (to online pornography, or online sex); and cyber-relationship addiction (to online relationships). Rather than becoming addicted to the medium *per se*, some users may develop an addiction to specific activities they conduct online (18).

Many negative health outcomes are associated with IA, regarding both general health and mental health, such as mood disorder, poor sleep quality, low self-esteem, impulsivity, and suicide (19–23), in addition, also an association with academic underachievement and vocational struggles was found (24). In Carli et al. (25) emerged that depression and symptoms of ADHD had a consistent correlation with problematic internet use, as well as obsessive-compulsive symptoms. Consequently, there has been growing interest in the role of risk and protective factors related to IA. To better understand the genesis and development of IA, and possible treatment targets, there are many factors to consider: individual characteristics, first of all, but also family- and school-related variables, and environmental variables too (5).

In this review, we focused on those examining religiosity, religious practice and spirituality as individual factors that might be related to IA, and on two specific aspects of IA, i.e., online gaming disorder, online gambling addiction. The population analyzed by the studies included in the review was that of adolescents and young adults (e.g., college students).

This study aimed to perform a systematic review of observational studies evaluating association between religiosity or spirituality and IA, the findings could be of interest for the design of intervention strategies for preventing adolescents and young adults from developing these conditions.

## Methods

### Search strategy

A comprehensive and systematic literature search was conducted in the Pub-Med and Scopus databases to identify observational (cross-sectional, cohort, and case-control) studies investigating the association between religiosity or spirituality (R/S) and IA. Using Boolean operators, the search process involved a search string obtained by combining the terms:

“religiosity” or “spirituality” or “religion” or “faith” or “religious^*^” with the terms “patholog^*^” or “problem^*^” or “addict^*^” or “compulsive” or “dependen^*^” or “disorder^*^,” “excessive” or “addict^*^” or “compulsive” or “abuse^*^” or “dependen^*^” or “disorder^*^,” and “internet” “video” or “computer” or “mobile phone^*^” or “cell phone^*^” or “cellular phone^*^” or “cellular telephone^*^” or “mobile telephone^*^” or “smartphone^*^” or “nomophobia” or “internet gaming,” “internet game^*^” or “online gaming” or “internet video game” or “internet gambling.” The search strings are reported in Appendix 1.

The records retrieved from the databases were imported in Endnote and duplicates were removed. The reviewer checked the search hits by reading the article titles and abstracts. If the results of a study were published more than once, only the most complete article was considered in the analysis. The author also checked the reference lists of the papers included in the review for any articles not already considered. Relevant studies cited in the reference lists of the publications already identified were tracked down as well.

In conducting this systematic review, the authors conformed to the Preferred Reporting Items for Systematic Reviews and Meta-Analysis (PRISMA) statement (26).

### Data extraction

The following data was extracted from each study: first author's name; year of publication; journal; study design; sampling method; characteristics of the study sample (e.g., age range); measures of outcome and of exposure; results; confounding factors; interactions; and the author's conclusions.

### Eligibility criteria

The studies included in the review had to meet the following inclusion criteria:

- a declared measure was used to assess religiosity or spirituality (considering spirituality as an aspect that can be experienced both outside and within a religious context (5), and characterized by a desire for transcendence, a sense of interconnection and a meaningful sense of life (6);
- a declared measure was used to assess different types of IA, as regards online gaming, and online gambling;
- a declared measure was used to assess the association between R/S and IA, or online gaming, or online gambling;
- published any time up until November 2021.
- written in English.

The studies excluded for the purposes of this review were:

- those involving samples that only included individuals who reported belonging to a religion;
- studies that did not distinguish between gambling on- and offline.

### Methodological assessment

One author judged the methodological quality of the studies using the Appraisal tool for Cross-Sectional Studies (AXIS) (27). The scoring system conforms to a “yes,” “no,” or “do not know/comment” design. The studies included in the review were categorized into four groups: >15, 10–15, 5–9, and ≤4 AXIS criteria met. Seven studies out of 13 met 10–15 AXIS criteria, six studies met more than 15 AXIS criteria; Appendix 2 outlines the methodological quality of the studies.

## Results

**Figure 1:**
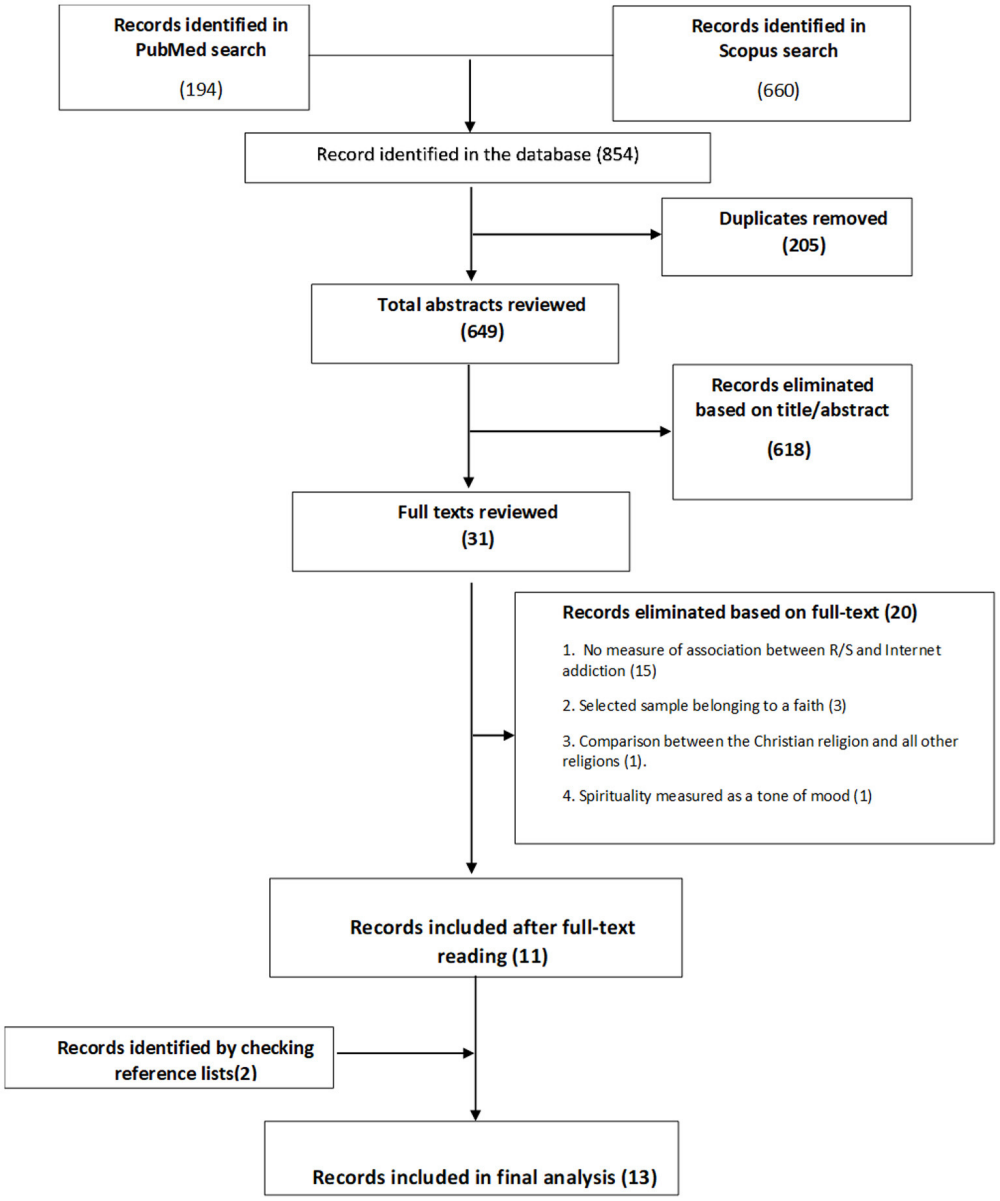
Flow chart of the article selection process. R/S, Religiosity/spirituality; IA, Internet addictions.

Two additional studies that met our inclusion and exclusion criteria were identified from the reference lists of the 11 selected articles, so the review was conducted on 13 articles in all. They were all were observational studies, and all cross-sectional. Figure 1 shows the flow chart of the article selection process.

The number of participants enrolled in each study ranged from 97 to 11,956. Six studies were conducted specifically on adolescents (28–33), two on young adults (34, 35), four on mixed samples of adolescents and young adults (36–39), and in only one study the sample consisted only of adults (40). The studies were conducted in different countries: two in the US (30, 35), two in Iran (36, 37), one in Turkey (39), one in China (28), one in Malaysia (31), two in Switzerland (34, 38), one in Poland (40), one in the Czech Republic (29), one in South Korea (33), and one was a multicenter study involving 10 countries: Austria, Estonia, France, Germany, Hungary, Ireland, Israel, Italy, Romania, and Slovenia (32).

The studies included in our review were published between 2012 and 2021.

Ten of the 13 articles selected investigated only religiosity as a predictive factor (28, 30–32, 34–37, 39, 40), while two considered both religiosity and spirituality (29, 33), and one only examined spirituality (38).

### Exposure measures

Only five studies used questions from validated questionnaires designed to assess R/S. In general, there was a marked variability in how exposure to R/S was measured. One study measured religiosity using the Religious Background and Behavior Questionnaire (34); one adapted the short form of the Santa Clara Strength of Religious Faith Questionnaire (31); one measured religiosity using a single question from the Global School-Based Pupil Health Survey (GSHS) and the European Values Study (EVS) about self-perception of being a religious person (32); one study measured religiosity using the Religious Belief Scale to ascertain high-school students' religious belief levels (39); one applied the Korean Brief God's Image Scale, which consists of 17 items covering five dimensions (answering, accepting, benevolent, presenting, and nurturing God's image), as a short form of Lawrence's God's Image Scale, which contains 72 items (33). Other studies used *ad hoc* questionnaires to investigate different domains of religiosity (28, 30, 35–37, 40), such as: praying, self-perception as a religious person, perception of the importance of religiosity, devotion, frequency of attending religious services, religious beliefs, and commitment (30). Finally, two studies (29, 33) investigated spirituality using the adjusted short version of the Spiritual Well-Being Scale (SWBS), and one (38) only measured spirituality by asking to what extent participants believed in a spiritual higher power, and in its supporting and judging roles. Only seven studies (31, 34–39) asked participants about their type of religion (if any).

### Outcome measures

The studies also varied in their approach to measuring the outcome. Twelve studies investigated IA (28, 30–33, 35–39); and three examined gaming (28, 29, 34); none specifically measured online gambling. In four studies (28, 36–38), severity of IA was assessed using Young's IA Test (IAT) (41). In two others, another version adapted by Young was also used to measure IA (32, 39). Other studies used various questionnaires: The Excessive Internet Use scale (29, 41), the Short Problematic Internet Use Test (SPIUT) (30, 42, 43), the Internet addiction scale (44) (adapted from an instrument validated by Charlton and Danforth) (31), the I-scale (Internet addiction scale developed by Kim et al.) (35). One study (33) used the Korean Smartphone Addiction Proneness Scale for Youth and Adults. One used an unvalidated *ad hoc* questionnaire (40).

To measure internet gaming disorder (IGD), one study used the C-SURF questionnaire, which included a modified version of the game addiction scale (GAS) (34). In one study IGD was investigated using questions about how often respondents spent playing video games, and for how long, and self-reported addiction to this type of behavior (40). Another study measured IGD with a single *ad hoc* question: “How many hours a day do you usually play games online in your free time?” (29).

Tables 1, 2 provide details of the observational studies identified and included in the review.

**Table 1:** Overview of studies. Materials and methods.

**References**	**Study design**	**Sample characteristics**	**Type of exposure**	**Type of outcome**
	**Sample methods**		**Measure of exposure**	**Measure of outcome**
Ahmadi and Saghafi (36)	**Study design:** Cross-sectional. **Sample methods:** 14- to 19-year-old students from high schools and secondary schools. The sample was selected using the multistage cluster sampling technique and final random selection. Twelve out of 30 provinces in Iran were selected in the first phase; then two schools were chosen from each province, taking geographical dispersion into account; then students at each school were randomly selected using systematic sampling for participation in the study.	Iran, Year of data collection not specified. Four thousand one hundred and seventy-seven participants, aged 14–19 years, Iranian high-school and secondary-school students.	**Type of Religion:** Islamic **Measure: Religiosity** was assessed with the question: “How much do you bind yourself to religious activities such as praying?” “How familiar are you with the Quran?” “How much do you consider yourself a religious person?” Each item was accompanied by five possible answers ranging from “not at all” to “very much,” and scoring from 0 to 4.	**Type of outcome:** IA **Measure of outcome:** The level of IA was assessed using Young's IA Test (IAT).
Ahmadi (37)	**Study design:** Cross-sectional. **Sample methods:** Students from high school and pre-college stage. Mean ± SD age of the participants was 16.6 ± 1.5 years. The sampling method was multi-stage cluster sampling performed in 13 provinces of Iran	Iran, Year of data collection not specified. Four thousand three hundred and forty-two students from high school and pre-college stage (both <18 and >18)	**Type of Religion:** Islamic **Measure:** Not specified, but religiosity was classified as: **Religious** devotion (compared to nothing) Very high, High, Usual, Low.	**Type of outcom**e**:** IA **Measure of outcome:** Young's IAT, Young's Internet Addiction Scale (IAS) (7,8) assessing different aspects related to IA.
Braun et al. (34)	**Study design:** Cross-sectional. **Sample methods:** Data derived from the Cohort Study on Substance Use Risk Factors (C-SURF), which aimed to detect factors associated with a risk of substance use and their related consequences in young recruits (>18). Recruitment year 2010–2011	Switzerland, years 2010–2011 Five thousand nine hundred and twenty-two participants.	**Type of Religion:** Roman Catholic, Protestants, Christian-Catholic, Christian-Orthodox, other Christian communities, Islam, Jewish, none (= without RD, or other church or religious communities) **Measure:** To measure the strength of belief in God, the C-SURF questionnaire used the first question of the **Religious** Background and Behavior Questionnaire. Participants chose between the following categories: atheist (“I do not believe in God”), agnostic (“I believe we cannot really know about God”), unsure (“I do not know what to believe about God”), spiritual (“I believe in God but do not practice religion”) or religious (“I believe in God and practice religion”)	**Type of outcome:** internet gaming disorder (IGD) **Measure of outcome:** participants reported the frequency of their on- and offline gaming (“never,” “a few times a year,” “one to three times a month,” “at least once a week,” “almost every day”) behavior. To detect IGD, the C-SURF questionnaire included a modified version of the game addiction scale (GAS)
Chen et al. (38)	**Study design:** Cross-sectional. **Sample methods:** Data were drawn from the first wave of Generation Free, a study including a sample of young people between 15 and 24 years old in post-compulsory education (high schools or professional schools) living in the canton of Fribourg in Switzerland. Year of recruitment 2014–2015	Switzerland, years 2014–2015 Five thousand one hundred and seventy-nine participants.	**Type of religion:** Catholic **Measure: Spiritual** beliefs were measured by asking to what extent participants believed: in a spiritual higher power (general spiritual belief); in it having a supporting role; and in it having a judgmental role. For each of these three variables, participants were divided into two subgroups, depending on whether they were certain or uncertain about their beliefs.	**Type of outcome:** IA **Measure of outcome:** The short version of the Internet Addiction Test (s-IAT) was used to assess the level of excessive internet use.
Lewczuk et al. (40)	**Study design:** Cross-sectional. **Sample methods:** The study was based on a nationally representative sample of Polish adults, aged between 18 and 69, and conducted online with a preregistered survey. Inclusion criteria: age 18+	Poland, year 2019 One thousand thirty-six participants.	**Type of religion:** not specified. **Measure: religiosity** was assessed with three statements: “I attend religious services regularly,” “Being religious is important to me,” and “I consider myself religious.”	**Type of outcome:** IA, social networking addiction, gaming addiction. **Measure of outcome:** frequency and time of using internet, social networking and video games, self-perceived behavioral addiction to internet use, social networking and online gaming measured with three items: “I am addicted to …,” “I have put off things I needed to do in order to …,” and “I feel depressed after…”
Lu et al. (28)	**Study design:** cross-sectional. The study was conducted in Anhui and Qinghai provinces (both Tibetan Chinese and Han Chinese) at two public middle schools in the Tibetan area of Qinghai province and two public middle schools in the urban areas of Anhui province. All students, aged between 11 and 20 years at the selected schools were invited to participate in the study. The survey was conducted anonymously and participation was entirely voluntary at both sites.	China, year 2017 One thousand three hundred and eighty-five participants.	**Type of Religion:** Not specified **Measure: Religiosity** was assessed with the question “Do you have any religious beliefs?” and the possible answers were Yes or No.	**Type of outcome:** IA **Measure of outcome:** Young's IAT
Malinakova et al. (29)	**Study design:** Cross-sectional. **Sample methods:** Data were obtained from a nationally representative sample of Czech adolescents participating in the 2014 Health Behavior in School-aged Children (HBSC) study. Inclusion criteria: 5, 7, and 9^*th*^ graders (corresponding to 11, 13, and 15 years of age) who had participated in the 2014 Health Behavior study.	Czech Republic, year 2014 Four thousand one hundred and eighty-two participants.	**Type of Religion:** not specified. **Measure:** RA (**religious** attendance) was measured as the frequency of attending church or religious sessions. **Spirituality** was measured using the adjusted shortened version of the Spiritual Well-Being Scale (SWBS)	**Type of outcome:** IA and online gaming. **Measure of outcome:** IA was measured with the Excessive Internet Use scale. Excessive online gaming was measured with the question: “About how many hours a day do you usually play games on a computer, games console, tablet (e.g., iPad), smartphone or other electronic device (do not count physical fitness games) in your free time.”
Atwood et al. (30)	**Study design:** Cross-sectional. **Sample methods:** Adolescents were recruited by advertising on social media (Facebook, Twitter, and Instagram) aimed at parents of adolescents between the ages of 14–18. The adolescents accessed the survey *via* a link provided by their parents. Inclusion criteria: adolescents between 14 and 18.	USA, year of data collection not specified. Ninety-seven participants.	**Type of Religion:** not specified **Measure: Religious** affiliation and commitment were assessed. Participants were asked to select their present religion from a large list of denominations, including “atheist” and “nothing in particular.” They were also asked to describe how committed they were to the teachings and practices of their religion or spiritual convictions, using a five-point Likert scale (0 = not at all committed, 4 = very committed).	**Type of outcome:** smartphone addiction. Problematic internet use (PIU). **Measure of outcome:** Short Problematic Internet Use Test (SPIUT). The SPIUT is a shortened version of the Compulsive Internet Use Scale (CIUS), and consists of six questions answered on a five-point Likert scale (0 = never, 4 = very often) to measure key factors of PIU: loss of control, preoccupation and salience, conflict, withdrawal symptoms, and coping, with composite scores being used to determine levels of PIU.
Charlton et al. (31)	**Study design:** Cross-sectional. **Sample methods:** Participants were from 16 randomly-selected schools in four states of Peninsular Malaysia. Inclusion criteria: school students, age between 15 and 17 years	Malaysia, year of data collection not specified. One thousand five hundred ninety-six participants.	**Type of religion:** Muslims, Christians, Buddhists, Hindus. **Measure:** The **religiosity** measure was adapted from the short form of the Santa Clara Strength of Religious Faith Questionnaire	**Type of outcome:** IA. **Measure:** The internet addiction scale was adapted from an instrument validated by Charlton and Danforth (44). Items were adapted by replacing the name of the computer game (44) study with a reference to the internet. For example, “Arguments have sometimes arisen at home because of the time I spend playing Asheron's Call” became “Arguments have sometimes arisen at home because of the time I spend on the internet.”
Koo et al. (35)	**Study design:** Cross-sectional. **Sample methods:** USA, year 2010. Data were collected through self-reported internet surveys. Only international undergraduate students aged between 12 and 19 years enrolled at a large private research institution in the US were recruited, by means of flyers, online advertisements, international student orientations, seminars and workshops for international students, and by word of mouth. Inclusion criteria: international undergraduate students.	USA, year 2010. One hundred fifty-seven participants.	**Type of Religion:** Protestant, Buddhist, Catholic, Muslim, Other **Measure:** participants were asked if they were **religious**; answers were dichotomized as “religious” or “not religious”	**Type of outcome:** IA. **Measure of outcom**e: The Internet Addiction Scale (I-Scale) was used to ascertain the presence and severity of IA.
Durkee et al. (32)	**Study design:** Cross-sectional. **Sample methods:** The study was conducted within the framework of the EU-funded Saving and Empowering Young Lives in Europe (SEYLE) project, a randomized controlled trial assessing interventions for the prevention of risky behavior in European school students. Adolescents were recruited from 178 randomly-selected schools at 11 study sites, in the following countries: Austria, Estonia, France, Germany, Hungary, Ireland, Israel, Italy, Romania, Slovenia, and Spain. A structured questionnaire was administered. Inclusion criteria: adolescents attending non-specialist public schools; the school authorities' agreement to participation; schools containing at least 40 pupils aged 15; schools with more than two teachers for pupils aged 15; no more than 60% of pupils of either sex; informed consent obtained from parents and pupils. Exclusion criteria: adolescents attending single-sex schools.	Austria, Estonia, France, Germany, Hungary, Ireland, Israel, Italy, Romania, Slovenia, and Spain; years 2009–2010 Eleven thousand nine hundred fifty-six participants.	**Type of Religion:** Not specified. **Measure: religiosity** was measured using questions from the Global School Based Pupil Health Survey (GSHS) and European Values Study (EVS). A single question was used, asking whether students perceived themselves as religious.	**Type of outcome:** Type of internet use: adaptive, maladaptive, and pathological **Measure of outcome:** Internet users were classified in three categories: adaptive, maladaptive (MIU) and pathological (PIU), based on their score in the Young Diagnostic Questionnaire for Internet Addiction (YDQ). Information was also obtained about the average number of hours spent online per day for non-essential purposes (e.g., not for schoolwork) and specific online activities.
Ekşi et al. (39)	**Study design:** Cross-sectional. **Sample methods:** Simple random sampling was used to form the study group, which consisted of high-school students attending several schools in Istanbul during the 2015–2016 academic year. Inclusion criteria: high-school students.	Turkey, Istanbul; years 2015–2016 Three hundred eighty-nine participants.	**Type of Religion:** Muslim **Measure:** The Religious Belief Scale, was used to ascertain high-school students' levels of **religious** belief. A Moral Maturity Scale was also used to measure individuals' moral maturity levels.	**Type of outcom**e: PIU. **Measure of outcome:** The Internet Addiction Scale was used.
Shim (33)	**Study design:** Cross-sectional. **Sample methods:** Age range from 12 to 14 years old. All participants were recruited online and offline from churches in Seoul and Gyonggi-do. Adolescents were divided into: those at high risk of smartphone addiction, those potentially at risk of smartphone addiction, and control participants.	Two hundred eighty-five participants from South Korea	**Type of Religion:** Christian **Measure:** Spiritua**l** wellbeing scale for Spirituality, and Korean Brief God's Image Scale developed from Lawrence's God's Image Scale	**Type of outcome:** Smartphone addiction **Measure of outcome:** Korean Smartphone Addiction Proneness Scale for Youth and Adults.

**Table 2:** Overview of study findings.

**References**	**Results**	**Confounders**	**Conclusions**
Ahmadi and Saghafi (36)	IA was significantly associated with subsequent variables: “Praying” has a *p* < 0.0001; “Considering yourself religious” *p* < 0.0001; “Familiar with Quran” *p* < 0.0001.		Family relationships was the most important factor related to IA, and religious beliefs was the second most important. All three religious variables seemed to correlate with the level of IA, and their influence was surprisingly more powerful than the family variable. Adolescents with stronger religious beliefs tended to be less affected by IA.
Ahmadi (37)	Odds ratios for Religious devotion (reference group nothing) were: Very high, OR 0.040 (*p* < 0.001); High, OR 0.571 (*p* 0.006); Usual OR 0.6 (*p* 0.033); Low, OR 0.65 (*p* 0.124)	Gender, age, mother's occupation, financial status, quality of family relationships	Students' lower levels of religious devotion were significantly associated with having IA. This study showed that IA is prevalent in Iranian adolescents, and that religious devotion is an independent factor associated with IA.
Braun et al. (34)	There was a significant inverse relationship between strength of belief in God and online gaming, GAS scores and the risk of excessive/addictive computer use. A stronger belief in God was associated with lower gaming frequency and lower scores on the game addiction scale. Practicing a religion was related to less frequent online and offline gaming. Finally, Christians gamed less frequently and had lower scores on the game addiction scale than participants without a religious denomination	Age and education level were computed as covariates, dichotomous variables and the RD as fixed factors.	Findings indicated that R/S might have a preventive effect against excessive/addictive computer gaming. These results could prove useful for developing preventive and therapeutic strategies for internet gaming disorder.
Chen et al. (38)	In the bivariate analysis, there was no statistically significant association between excessive internet use and belief in a spiritual higher power (*p* = 0.2); in the logistic regression analysis, excessive internet use was not associated with belief in the supporting role of a spiritual higher power (*p* = 0.067). The risk of internet addiction was significantly increased by certainty about the judgmental role of a spiritual higher power (OR: 1.74, 95% CI: 1.38–2.20, *p* < 0.001).	Age, gender, language, and place of birth	The study found that spiritual belief could increase the risk of excessive internet use.
Lewczuk et al. (40)	Religiosity did not predict the prevalence of addiction to internet use, social networking, or online gaming.	Gender, age, “moral incongruence” regarding behavioral addiction to internet use, social networking and online gaming (measured with one item: “I believe that… is morally wrong”) and time and frequency of engagement in a particular addictive behavior.	Religiosity was uniquely, but weakly, connected to addiction to pornography, but not to other types of addictive behavior, such as internet addiction and others.
Lu et al. (28)	Multiple logistic regression analysis revealed that religious beliefs were positively associated with IA. Religious beliefs: OR 1.5 (*p*-value 0.04).	Age, sex, number of children in the family, living conditions (living with family/others), perception of personal health and weight (bad/fair/good), pressure of studies (little/fair/high), and relationships with classmates, teacher and family (poor/fair/good).	A positive association was found in this preliminary study between religious beliefs and IA.
Malinakova et al. (29)	Compared to non-attending and non-spiritual respondents, respectively, both attending respondents and spiritual respondents were less likely to play computer games excessively, with odds ratios (ORs) ranging from 0.6 (95% confidence interval 0.5–0.8) to 0.92 (0.9–0.99). “Only attending” and “only spiritual” respondents were more likely to use the internet excessively, but this was not the case for those who were both attending and spiritual.	Gender, age, socioeconomic status, and perceived family support.	The combination of attending religious activities and being spiritual was protective against excessive internet use. RA and spirituality were associated with a more active way of spending leisure time. This suggests that increasing secularization might lead to further unfavorable changes in adolescents.
Atwood et al. (30)	Teens with lower levels of religious commitment [(beta) = −0.23, *p* < 0.05] spent slightly more time online each day, even after controlling for their primary source of internet access. Participants who reported high levels of religious commitment tended to spend less time on the internet.	Age, gender, and family structure.	Religious commitment was a major factor that, in most cases, seemed to outweigh the impact that teens' primary source of internet access had on their behavior. Among the participants in this study, religiosity was more closely related to the behaviors examined than parental mediation attempts or the device teens used to access the internet. Teens who were highly religious and who had a strong attachment to their parents were less likely to use the internet in problematic ways.
Charlton et al. (31)	Correlations (Pearson's *r*) between IA and religiosity for Muslim: −0.05 for males, −0.11 with *P* < 0.01 for females. Correlations (Pearson's *r*) between IA and religiosity for Buddhists: −0.07 for males, −0.21 with *P* < 0.001 for females. Correlations (Pearson's *r*) between IA and religiosity for Hindu: 0.05 for males, −0.39 with *P* < 0.001 for females. Correlations (Pearson's *r*) between IA and religiosity for Christians: −0.005 for males, −0.28 with *P* < 0.05 for females.		Religiosity might have benefits in reducing adolescents' susceptibility to internet-related addictions, but this might only apply to females. In Malaysia at least, more religious females (but not males) are less likely to become addicted to internet.
Koo et al. (35)	The IA scores of religious international students were significantly lower (M = 58.33, SD = 17.71) than those of non-religious international students (M = 61.84, SD = 19.26), *t*-test= 3.51, *p* < 0.05.		Students who were religious had significantly lower scores for IA addiction than non-religious peers.
Durkee et al. (32)	In both models, religiosity was not correlated with IA. IA total score and IA categories were not associated with religiosity in the linear regression model or the multinomial regression model.	Age, gender, internet accessibility, population distribution, household composition, adolescent or parent(s) born in another country, parental involvement and unemployment, peer relationships.	Religiosity was not a significant factor for either MIU or PIU.
Ekşi et al. (39)	No significant relationship was found between problematic internet use and religious belief (*r* = −0.029, *p* > 0.01). When examining the regression coefficients, only the variable of moral maturity emerged as a significant predictor of problematic internet use. Religious belief did not significantly contribute to problematic internet use	Moral maturity	Moral maturity would seem to be a significant predictor of high-school students' problematic internet use, while religious belief showed no significant association with it.
Shim (33)	God's image and spirituality differed significantly between the groups divided by the degree of smartphone addiction. The significant difference in smartphone addiction between the high-risk group, potential-risk group, and normal control group in terms of God's image and spiritual wellbeing was confirmed (*p* < 0.0005). The accepting God's image score in the high-risk group was significantly lower than that of the normal control group. The religious wellbeing and existential wellbeing scores of the potential-risk group were significantly lower those of the normal control group. The high-risk group's existential wellbeing score was significantly lower than that of the normal control group.		From a research perspective, this study revealed the importance of spirituality in relation to the level of smartphone addiction by exploring the unique characteristics of God's image and spiritual wellbeing among addicted groups. The results showed that the group at high risk of smartphone addiction showed low levels of spiritual wellbeing and a positive image of God compared with the potential-risk and control groups. A negative image of God closely correlated with smartphone addiction, as reported in previous research.

Among the 12 studies that investigated IA in relation to religiosity, six found an inverse association between them (30, 31, 33, 35–37), three found no evidence of any association (32, 39, 40), and one identified a direct association (28). The only study that investigated both religiosity and spirituality (29) was conducted on adolescents in the Czech Republic. It found that attributing more value to both religiosity and spirituality coincided with a lower probability of IA, but attributing more value only to spirituality or only to religiosity (assessed here in terms of the amount of religious practice) was associated directly with a greater IA.

Of the eight studies showing a protective effect of religiosity against IA (29–31, 33–37), six were conducted on adolescents: two in Iran (36, 37), one in the Czech Republic (29), one in the US (30), one in Malaysia (31), and one in South Korea (33). The other two studies were conducted on particular samples of adults: young army recruits in Switzerland in one case (34), and international undergraduate students at a research institution in the US in the other (35).

Three studies (32, 39, 40) found no evidence of any association between religiosity and IA: one was a study on a nationally-collected representative sample of adults in Poland (40), one involved young adolescents in Turkey (39); and one (32) was conducted on a large sample of more than 10,000 adolescents from numerous European countries (e.g., Italy, Spain, Germany, France and Austria, among others). Finally, a study conducted in China (28) on a sample of adolescents found religiosity directly associated with IA: this study measured religiosity with a single, *ad hoc* question.

The only study investigating spirituality unrelated to any religion found a direct association with IA (38). It was conducted on young Swiss people in further, post-compulsory education. While it found no evidence of any association between belief in a “spiritual higher power” or in the “supporting role of a higher power” and IA, but it also showed that the risk of IA was increased significantly by the “judgmental role” of such a higher power.

Two out of three studies investigating IGD in particular found an inverse association between higher levels of religiosity and IGD (29, 34), and one found no such association (40). No studies specifically investigating the association between online gambling and R/S came to light.

## Discussion

This systematic review investigated the association between religiosity and the emerging internet addictions in adolescents and young adults. Most of the studies reviewed found lower levels of internet addiction associated with religiosity (30, 31, 33, 35–37), one study found lower levels of internet addiction associated with religiosity only when combined with spiritual belief (29), while more than one in four found no statically significant association (32, 39, 40), and only one found a direct association (28). The study only investigating spirituality (38) found a growing attitude toward internet addiction in the spiritual group. Among three studies investigating online gaming addiction in particular, one found an inverse association between religiosity and gaming (34), one found an inverse association between religiosity or spirituality and gaming (29) and one found no association (40). Our database search identified no studies specifically investigating religiosity or spirituality and online gambling.

Six out of 11 of the studies reviewed (30, 31, 33, 35–37) found that religiosity acted as a protective factor against IA. In particular, Atwood's study (30) on a sample of students in the USA found religious commitment a major factor that seemed to outweigh the impact that teens' primary source of internet access had on their behavior. Teens who were strongly religious and had a strong attachment to their parents were less likely to use the internet or smartphones in problematic ways. Two Iranian studies included in our review also found that the influence of religious beliefs was more powerful than the family variable, and adolescents with stronger religious beliefs tended to be less affected by IA (36, 37).

These results are supported by a recent study, which found that a faith-based and spiritual approach helps people to feel grounded, calm, resilient, and present in difficult environments (45). Attributing a religious dimension to existence is often related to optimism, generosity and gratitude, and to the opportunity to lend meaning to life (46). On the other hand, excessive internet use has been described as a coping strategy, part of a psychological and behavioral effort to reduce the stress caused by external or internal events experienced as physically or mentally demanding (47). Religious people could consequently be less likely to resort to escaping into a self-created virtual online world. In agreement with Koo et al. (35), the present findings could also be explained by a better self-control and greater ability to delay gratification in religious adolescents and young adults, which could weaken any dependence on addictive behavior such as an excessive internet use. It has been reported, moreover, that there is a strong positive correlation between loneliness and Internet addiction among adolescents (48). Van Olphen et al. (49) claims that being integrated in a community of faith is an important source of social support.

None of studies included in our review showed an inverse association between spirituality and IA. On the contrary, one study (29) found that spirituality without the combined religiosity can constitute a risk factor for IA and a study conducted on Swiss adolescents (38) found that the risk of excessive internet use was increased particularly by the idea of a higher spiritual power with a “judgmental role,” in the sense of a negative image as a punisher. Regarding this finding, it seems appropriate to consider the role of spiritual beliefs in relation to the separation-individualization process and the transition from adolescence to adulthood. “Rule-breaking” behavior is common among adolescents during the process of separation-individualization (50). Chen et al. (38) wrote that their findings also suggest that the relationship between the perceived “judgmental role” of a spiritual power and an adolescent's excessive internet use might not be simply causal, but also interactive, and modified (and motivated) by a normal process of adolescent separation-individualization. Furthermore, aspects such as self-actualization and individualism, inherent to some types of spirituality could play an important role (51, 52) and, as suggested by Sussman et al. (53), adolescents search for meaning, purpose and identity, and this desire for transcendence might lead them to addiction behavior. Shim (33) noted that this somewhat counterintuitive point is still being explored by various research groups, and highlighted the complicated relationships between spirituality and addictive behavior more generally. As a consequence—according to Grubbs and Grant (8), the links between religion/spirituality and internet-related addictions are much less clear than those between religion/spirituality and other addictive behavioral disorders. These mixed results point to the need for further work to clarify the nuanced relationship between religion/spirituality and risky online behaviors.

Two (29, 34) of the three studies that looked at the association between religiosity and problematic gaming behavior found that higher levels of religiosity coincided with lower levels of gaming in general, and a lower risk of addictive gaming tendencies. In a study (34) on a sample of young Swiss army recruits, Braun found that a stronger belief in God was associated with a lower gaming frequency and smaller scores on a game addiction scale. A Czech study on adolescents (29) found that respondents who attended religious services and were more spiritual were less likely to make excessive use of the internet or computer games. Malinakova et al. (29) hypothesized that, in families with a greater religiosity/spirituality, parents tend to keep a more careful eye on adolescents' behavior and risk of addictions; their religiosity/spirituality may also promote their child's internalization of adult behavioral norms. Plante et al. (54) found that using online games to avoid stress is a predictor of symptoms of gaming addiction. This points to the idea that addicted gamers use gaming as a means of escape from reality (55), whereas previous research (34, 56–58) underscored how a belief in God may represent a strategy for coping with stressful life events. When something stressful happens, people who are more religious resort to a more functional style of coping with difficulties, finding support in the religious communities to which they belong (59), or adopting behaviors such as prayer or gestures that carry an intrinsic meaning for them, reducing stress and promoting resilience. Kim et al. (60) also suggested that religious people may not sufficiently gratified by immersing themselves in a virtual world. It seems difficult to explain the protective effect of religious practice against online gaming, however (4). Lewczuk et al. (40) found no statistically detectable links between religiosity and IGD in a Polish adult sample. On the other hand, they found that adolescents experiencing a “moral incongruence”—in the sense of a sense of distress connected with others' disapproval when they engaged in their addictive behavior—could be more at risk of IGD. Previous studies had already suggested that a problem of “moral incongruence” related to high levels of religiosity could predict addictive behavior (61).

### Strength and limitations

This is the first study (to our knowledge) to systematically review the evidence of the association between people's R/S and the risk of IA and other internet-related addictive behavior. This review has a number of limitations to consider, however. First, the various studies reviewed used different tools to measure both religiosity and IA, preventing any quantitative meta-analysis. They were also all cross-sectional, making it impossible to draw any causal inferences. That said, the particular nature of the exposure examined prevents the use of a more robust study design, such as a randomized and controlled trial, to clarify the possible existence of a cause-and-effect relationship, and possibly identify which components of religiosity have a protective role in discouraging the onset of IA. As a consequence, the results of these observational studies remain important for the purposes of better adapting interventions and shaping the social context to prevent IA, especially in adolescents and young adults.

## Conclusion

The findings of this review point to a protective role for religiosity against internet-related addictions. These results could be of the utmost importance when considering the strategies needed to prevent such addictions, and the environmental and social settings in which in adolescents and young adults are at greatest risk.

## Author contributions

AB conceptualized the study and approved the final manuscript as submitted. LM and FD conducted literature searches and provided summaries of previous research studies. FD and AB wrote the first draft of the manuscript. All authors contributed to and have approved the final manuscript.

## Conflict of interest

The authors declare that the research was conducted in the absence of any commercial or financial relationships that could be construed as a potential conflict of interest.

## Publisher's note

All claims expressed in this article are solely those of the authors and do not necessarily represent those of their affiliated organizations, or those of the publisher, the editors and the reviewers. Any product that may be evaluated in this article, or claim that may be made by its manufacturer, is not guaranteed or endorsed by the publisher.

## Abbreviations

C-SURF: Cohort Study on Substance Use Risk Factors
EVS: European Values Study
GAS: Game Addiction Scale
GSHS: Global School Based Pupil Health Survey
IA: Internet addiction
IAS: Young's Internet Addiction Scale
IAT: Young's IA Test
IGD: Internet gaming disorder
MIU: maladaptive Internet use
OR: odds ratio
PIU: problematic Internet use
R/S: religiosity/spirituality
s-IAT: short version of the Internet Addiction Test
SPIUT: Short Problematic Internet Use Test
WHO: World Health Organization
YDQ: Young Diagnostic Questionnaire for Internet Addiction
RD: religious denomination
RA: religious attendance.
