# Supplementary material for: Association between religiosity or spirituality and internet addiction: A systematic review
Source: Front Public Health. 2022 Dec 1;10:980334. doi: 10.3389/fpubh.2022.980334 (PMC9751319; doi:10.3389/fpubh.2022.980334)
Supplement: Supplementary file 1 [file Table_1.docx]

**Appendix 1**

**Pubmed search string**

(Religion [Title/Abstract] OR spirituality [Title/Abstract] OR religiosity [Title/Abstract] OR faith [Title/Abstract] OR religious*[Title/Abstract] OR religiousness [Title/Abstract]) AND (patholog* [Title/Abstract] OR problem* [Title/Abstract] OR addict* [Title/Abstract] OR compulsive [Title/Abstract]OR dependen* [Title/Abstract] OR disorder* [Title/Abstract] OR excessive [Title/Abstract] ) AND (video [Title/Abstract] OR computer [Title/Abstract]OR internet [Title/Abstract]OR mobil phone* [Title/Abstract]OR cell phone* [Title/Abstract]OR cellular phone* [Title/Abstract] OR cellular telephone* [Title/Abstract]OR mobile telephone* [Title/Abstract] OR smartphone* [Title/Abstract] OR nomophobia [Title/Abstract]OR internet gaming [Title/Abstract] OR internet game* [Title/Abstract]OR online gaming [Title/Abstract]OR internet video game [Title/Abstract]OR internet gambling [Title/Abstract] ) AND English [lang]

**Scopus search string**

( TITLE-ABS ( religion ) OR TITLE-ABS ( spirituality ) OR TITLE-ABS ( religiosity ) OR TITLE-ABS ( faith ) OR TITLE-ABS ( religious* ) OR TITLE-ABS ( religiousness ) AND TITLE-ABS ( patholog* ) OR TITLE-ABS ( problem* ) OR TITLE-ABS ( addict* ) OR TITLE-ABS ( compulsive ) OR TITLE-ABS ( dependen* ) OR TITLE-ABS ( disorder* ) OR TITLE-ABS ( excessive ) AND TITLE-ABS ( video ) OR TITLE-ABS ( computer ) OR TITLE-ABS ( internet ) OR TITLE-ABS ( mobil AND phone* ) OR TITLE-ABS ( cell AND phone* ) OR TITLE-ABS ( cellular AND phone* ) OR TITLE-ABS ( cellular AND telephone* ) OR TITLE-ABS ( mobile AND telephone* ) OR TITLE-ABS ( smartphone* ) OR ( nomophobia ) OR TITLE-ABS ( internet AND gaming ) OR TITLE-ABS ( internet AND game* ) OR TITLE-ABS ( online AND gaming ) OR TITLE-ABS ( internet AND video AND game ) OR ( online AND gambling ) ) AND ( LIMIT-TO ( LANGUAGE , "English" ) )
