# Supplementary material for: Association between religiosity or spirituality and internet addiction: A systematic review
Source: Front Public Health. 2022 Dec 1;10:980334. doi: 10.3389/fpubh.2022.980334 (PMC9751319; doi:10.3389/fpubh.2022.980334)
Supplement: Supplementary file 2 [file Table_2.docx]

| **Appendix 2: Methodological appropriateness of the studies, Appraisal tool for Cross-Sectional Studies (AXIS)** | | | | | | | | | | | | | |  |  |  |  |  |  |  |
| --- | --- | --- | --- | --- | --- | --- | --- | --- | --- | --- | --- | --- | --- | --- | --- | --- | --- | --- | --- | --- |
|  | **1** | **2** | **3** | **4** | **5** | **6** | **7** | **8** | **9** | **10** | **11** | **12** | **13** | **14** | **15** | **16** | **17** | **18** | **19** | **20** |
| **Ahmadi et al. (28) 2013 Cyberpsychology behavior, and social networking** | YES | YES | YES | YES | YES | YES | NO | YES | NO | YES | YES | YES | ND | NO | YES | YES | YES | NO | YES | YES |
| **Ahmadi (29) 2013 Acta Medica Iranica** | YES | YES | YES | YES | YES | YES | NO | YES | NO | YES | YES | YES | ND | NO | YES | YES | YES | NO | NDis | NS |
| **Braun et al. (30) 2015 J Relig Health** | YES | YES | YES | YES | YES | YES | NO | YES | YES | YES | YES | YES | ND | NO | YES | YES | YES | YES | NDis | NS |
| **Chen et al. (31) 2018 Int J Adolesc Med Health** | YES | YES | YES | YES | YES | YES | NO | YES | NO | YES | YES | YES | ND | NO | YES | YES | YES | YES | YES | YES |
| **Lewczuk et al. (32) 2020 Society for the Study of Addiction** | YES | YES | YES | YES | YES | YES | NO | YES | NO | YES | YES | YES | ND | NO | YES | YES | YES | YES | YES | YES |
| **Lu et al. (33) 2018 Psychiatry Research** | YES | YES | YES | YES | YES | YES | NO | YES | YES | YES | YES | YES | ND | NO | YES | YES | YES | YES | NDis | YES |
| **Malinakova et al. (34) 2018 PLoS ONE** | YES | YES | YES | YES | YES | YES | YES | YES | YES | YES | YES | YES | ND | NO | YES | YES | YES | YES | NDis | NS |
| **Atwood et al. (35) 2017 North American Journal of Psychology Utah State University** | YES | YES | YES | YES | YES | YES | NO | YES | YES | YES | YES | YES | ND | NO | YES | YES | YES | YES | NDis | NS |
| **Charlton et al.2012 (36)  Information,Communication &amp; Society** | YES | YES | YES | YES | YES | YES | NO | YES | YES | YES | YES | YES | ND | NO | YES | YES | YES | YES | NDis | NO |
| **Koo et al. (37) 2021 Journal of International Students** | YES | YES | YES | YES | YES | YES | NO | YES | NO | YES | YES | YES | ND | YES | YES | YES | YES | YES | NDis | NS |
| **Durkee et al. (38) 2012 Addiction Sweden** | YES | YES | YES | YES | YES | YES | YES | YES | YES | YES | YES | YES | ND | YES | YES | YES | YES | YES | YES | YES |
| **Ekşi et al. (39) 2017 Addicta: The Turkish Journal on Addictions. Turkey.** | YES | YES | YES | YES | YES | YES | NO | YES | YES | YES | YES | YES | ND | NO | YES | YES | YES | NO | NDis | NO |
| **Shim J. (40) 2019 J Relig Health** | YES | YES | YES | YES | YES | YES | YES | YES | YES | YES | YES | YES | ND | NO | YES | YES | YES | YES | YES | YES |
| Abbreviations:  ND – not described;  NDis – not disclosed;  NS – not stated |  |  |  |  |  |  |  |  |  |  |  |  |  |  |  |  |  |  |  |  |
